# The Impact of an Inactivated Hepatitis A Vaccine with One Dose in Brazil: A Retrospective Time-Series

**DOI:** 10.3390/vaccines9040407

**Published:** 2021-04-20

**Authors:** Ana Luiza Bierrenbach, Yoonyoung Choi, Paula de Mendonça Batista, Fernando Brandão Serra, Cintia Irene Parellada, Guilherme Silva Julian, Karina Nakajima, Thais das Neves Fraga Moreira

**Affiliations:** 1 Instituto de Ensino e Pesquisa, Hospital Sírio-Libanês, São Paulo 01308-050, SP, Brazil; 2 Merck & Co., Inc., Kenilworth, NJ 07033, USA; yoon.young.choi2@merck.com; 3 MSD Brazil, São Paulo 04717-004, SP, Brazil; paula.de.mendonca.batista@merck.com (P.d.M.B.); fernando.brandao.serra@merck.com (F.B.S.); cintia.parellada@merck.com (C.I.P.); thais.moreira@merck.com (T.d.N.F.M.); 4 IQVIA Brazil, São Paulo 04719-002, SP, Brazil; guilherme.julian@iqvia.com (G.S.J.); karina.nakajima@iqvia.com (K.N.)

**Keywords:** hepatitis A, national immunization program, interrupted time-series analysis

## Abstract

Background: In 2014, a recommended one-dose of inactivated hepatitis A vaccine was included in the Brazilian National Immunization Program targeting children 12–24 months. This decision addressed the low to intermediate endemicity status of hepatitis A across Brazil and the high rate of infection in children and adolescents between 5 and 19 years old. The aim of the study was to conduct a time-series analysis on hepatitis A incidence across age groups and to assess the hepatitis A distribution throughout Brazilian geographic regions. Methods: An interrupted time-series analysis was performed to assess hepatitis A incidence rates before (2010–2013) and after (2015–2018) hepatitis A vaccine program implementation. The time-series analysis was stratified by age groups while a secondary analysis examined geographic distribution of hepatitis A cases. Results: Overall incidence of hepatitis A decreased from 3.19/100.000 in the pre-vaccine period to 0.87/100.000 (*p* = 0.022) post-vaccine introduction. Incidence rate reduction was higher among children aged 1-4 years old, with an annual reduction of 67.6% in the post-vaccination period against a 7.7% annual reduction in the pre-vaccination period (*p* < 0.001). Between 2015 and 2018, the vaccination program prevented 14,468 hepatitis A cases. Conclusion: Our study highlighted the positive impact of a recommended one-dose inactivated hepatitis A vaccine for 1–4-years-old in controlling hepatitis A at national level.

## 1. Introduction

Hepatitis A is an acute liver disease caused by hepatitis A virus (HAV) infection. The prevalence of hepatitis A is closely associated with socioeconomic development and sanitation policies that ensure access to clean water, and adequate conditions for personal hygiene [1]. Although HAV transmission occurs primarily via exposure to contaminated food and water, many outbreaks of HAV infection have been associated with drug abuse and sexual behaviors, including men having sex with men (MSM) [2,3]. The clinical manifestations of hepatitis A are age-related, with less than 30% of patients under 6 years of age presenting with symptoms, while in adults more than 70% manifest symptoms such as fever, malaise, abdominal discomfort, nausea, vomiting, and jaundice [4]. HAV infection is usually self-limiting, however, 1% of cases evolve to acute liver failure, with the highest rates observed in older adults (>40 years old) or in patients with underlying chronic liver disease [5]. 

Between 1999 and 2015, HAV infection comprised 31% of all viral hepatitis reported in Brazil, with the North and Northeast regions accounting for 57% of all confirmed cases of hepatitis A and presenting the highest age-standardized mortality rate [6,7]. Since 2006, the overall incidence rate of HAV infection in Brazil has shown a downward trend, from 9.1/1,000,000 inhabitants to 3.1/1,000,000 in 2013 [7]. This considerable reduction is likely to be closely associated with improvements in water supply and sanitation. It is also important to note that a national serological survey conducted between 2004 and 2009 demonstrated that the country was in epidemiological transition, presenting two distinct patterns across the Brazilian regions. The North, Northeast and Midwest regions showed an intermediate endemicity pattern, in which 56.0–67.5% of children and adolescents aged 5–19 years were seropositive, compared to only 34.5–37.7% in the South and Southeast regions [8,9]. Increased outbreaks were reported across the country, most of them affecting the children and adolescents in this age group. In this situation, the World Health Organization (WHO) recommends integration of universal HAV immunization schedule for children ≥ 1 year old to reduce the number of overall hepatitis A and control outbreaks [10]. 

Most hepatitis A immunization schedules traditionally involve two doses of an inactivated hepatitis A vaccine, given 6-18 months apart for complete and life-long immunization [11]. Alternatively, a single-dose inactivated hepatitis A vaccine immunization schedule can be put into practice, offering seemingly comparable effectiveness, and easier implementation than the classic two-dose schedule. A universal single-dose childhood vaccination program has been adopted in some Latin American countries due to a competitive National Immunization Program (NIP) fund allocation. In July 2014, an inactivated hepatitis A vaccination program with a single-dose scheme targeting children between 12 and 24 months old was included in the Brazilian NIP, and expanded to cover children under 5 years old in 2017. This was based on experience in Argentina, where a decrease in mean incidence rate from 66.5/100,000 in 2000–2002 to 7.9/100,000 in 2006–2011 was reported after a single-dose program implementation in 2005, as well as through further endorsement in the 2012 WHO position paper on hepatitis A vaccines [10]. 

Previous data on the impact of universal hepatitis A vaccination in Brazil have demonstrated a cumulative 85.5% reduction in the total number of hepatitis A cases reported between 2014 and 2016 with vaccination coverage ranging from 60.1% to 97.1% [12,13]. The reduction was more prominent among children under 5 years old suggesting a direct vaccination effect. Additionally, the sharp decline in hepatitis A cases in children between 5 and 14 years old indicated the herd immunity phenomenon. Despite the importance of these data, only a descriptive analysis was performed, and the reduction of hepatitis A cases did not account for the baseline downward trend observed in the pre-vaccination period. It is, therefore, unknown whether the impact of the NIP on prevention of hepatitis A persists when pre-vaccination period effects are offset. It would also be useful to explore if the reduction in hepatitis A cases was consistent across different age groups and geographic locations after the universal vaccine introduction in order to identify potential outbreak triggers. Finally, it is important to measure the direct effect of the vaccination program by estimating the averted hepatitis A cases after the vaccine introduction. 

The aim of the current study was to assess the effect of universal childhood vaccination on hepatitis A incidence across age groups by conducting an interrupted time-series analysis. Additionally, we assessed the hepatitis A distribution throughout Brazilian geographic regions before and after the vaccination program implementation.

## 2. Materials and Methods 

### 2.1. Study Design and Population

An interrupted time-series analysis was performed to assess changes in hepatitis A incidence rate in Brazil from 2010 to 2018. An inactivated hepatitis A vaccine (Vaqta™ Ped/Adol vaccine, 25U of inactivated HAV antigen—Merck & Co., Inc., Kenilworth, NJ, USA) was incorporated in the Brazilian NIP in July 2014. We analyzed the period before (2010–2013) and after (2015–2018) the introduction of hepatitis A vaccine in the NIP, excluding the year 2014, considered as a transition period. The time-series analysis was conducted for the following age groups: 1–4 years old (target immunization population); 5–14 years old; 15–39 years old; over 40 years old. A secondary time-series analysis was performed to account for the outbreak among young adults in the Southeast region between 2017 and 2018 [14]. To project an outbreak-free scenario, this analysis considered the age groups of 15–39 years and over 40 years old, in addition to the overall population, and excluded data from the main affected states, São Paulo and Rio de Janeiro. Additionally, the hepatitis A incidence rate was analyzed for the five geographic regions of Brazil: North, Northeast, Center-West, Southeast, and South. The total number of fulminant hepatitis cases was also assessed in the pre- and post-vaccination period. 

### 2.2. Data Sources

Data were extracted from the Brazilian National Epidemiologic Surveillance System (SINAN), made available by the Brazilian IT Department of Public Healthcare System (DATASUS). Since 1996, all viral hepatitis cases (suspicious and confirmed) are classified as diseases of compulsory notification, and data are publicly available in the SINAN database. We extracted all confirmed hepatitis A cases reported between January 2010 and December 2018 in Brazil, defined as those with positive IgM anti-HAV or those that have detected HAV RNA or those that meet the clinical criteria and had contact with a laboratory-confirmed hepatitis A prior to onset of symptoms (e.g., household or sexual) or those that have died due to hepatitis A, as registered in the national mortality information system. Data were stratified according to age group and geographic region. Additionally, all cases classified in SINAN as fulminant hepatitis were extracted. 

Data on vaccination coverage were extracted from the NIP Information System (SI-PNI), also made available by DATASUS. The estimates of vaccination coverage in Brazil are mainly obtained through administrative method, where the numerator is defined by the number of vaccine doses administered that are registered in the public health units, and the denominator is defined by the population census estimates. For both vaccination coverage and incidence rate calculations, the yearly population estimate was obtained from the census available in the Brazilian Institute of Geography and Statistics (IBGE) database.

### 2.3. Data Analysis

Vaccination coverage was defined as the total number of immunized in the target-population per total number of target-population, within the region and period of analysis. Incidence rate was the sum of all new cases of hepatitis A in the period of analysis per 100,000 inhabitants, using total population for each age group and geographic region according to the year of analysis. 

A univariate Poisson regression model was used to compare the mean hepatitis A incidence rate in the pre-vaccination (from January 2010 to December 2013) and post-vaccination periods (from January 2015 to December 2018) across Brazilian regions. The overall mean incidence of fulminant hepatitis was also compared between pre- and post-vaccination periods. 

For the interrupted time-series analysis, a generalized negative binomial linear model with linked logarithmic function was used to predict counterfactual hepatitis A cases that would have been expected in the absence of vaccination for the post-vaccination period. To convert the monthly cases of hepatitis A into a rate, the logarithmic function of the population divided by 100,000 was included as an offset variable, adjusting for any potential changes in the population over time. Data from the transition period (2014) were excluded. The vaccination effect on hepatitis A incidence rates was calculated per year after exponentiation of model coefficients (slope change) of both pre-vaccination (trend without vaccination effect) and post-vaccination (trend with vaccination effect) period. The immediate effect was the level change observed immediately after the vaccine introduction. The vaccine impact was assessed as the level change and as the counterfactual comparison with the post-vaccination trend. A simulation bootstrap with 1000 replications was used to estimate the total number of averted hepatitis A cases, represented as median and percentiles 2.5% and 97.5%.

## 3. Results

### 3.1. Vaccination Coverage

**Figure 1:**
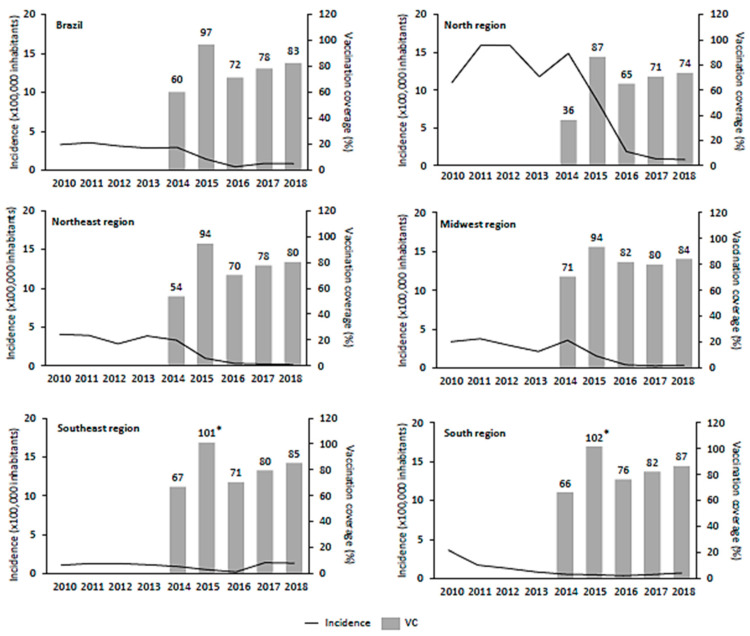
Incidence rate vs. vaccination coverage in different Brazilian geographic regions. * VC exceeded 100% coverage due to estimation errors in the population census.

The hepatitis A vaccine was implemented in July 2014 and in that year, the overall coverage was 60.1%. In 2015, adherence to the hepatitis A vaccination program was high in all regions, and the expected national target coverage of 95% was surpassed (97.1%). In the following years, however, the national vaccination coverage decreased to 71.6%, 78.5%, and 82.7%, in 2016, 2017, and 2018, respectively. Throughout the period of analysis (2014–2018), the mean vaccination coverage in Brazil was 78.0%. The North region had the lowest vaccination coverage throughout the study period (Figure 1). In some cases, the vaccination coverage exceeded 100% coverage, due to estimation errors in the population census. 

### 3.2. Hepatitis A Cases and Incidence

Between 2010 and 2018, 38,187 cases of hepatitis A were reported across all ages. Most cases occurred in children 5–9 years, comprising 29.6% of all cases during the study period. The vaccination target population (1–4 years old) presented a total of 4678 (12.3%) hepatitis A cases within the period of analysis (Table A1). The North region had the highest number of cases, with a total of 13,796 (36.1%) between 2010 and 2018. The overall incidence rate of hepatitis A reduced significantly after the vaccine program implementation, ranging from 3.18/100,000 in 2010 to 0.87/100,000 in 2018 (*p* = 0.022) (Table 1). The greatest impact of hepatitis A vaccination was observed in the North region, where the incidence rate decreased from 13.69/100,000 in the pre-vaccination period to 3.04/100,000 in the post-vaccination period (*p* < 0.0001) (Figure 1, Table 1). In this region, a dramatic decrease in the hepatitis A incidence was observed between 2015 and 2016, with a sustained low rate afterward. The incidence rate also decreased significantly in the Northeast and Midwest regions after vaccine introduction into the NIP, ranging from 3.67 to 0.42/100,000 (*p* = 0.001) and 3.06 to 0.66/100,000 (*p* = 0.013), respectively. For the South and Southeast regions, despite the reduction in the hepatitis A incidence, no statistical differences were observed in the comparison between pre- and post-vaccination period.

### 3.3. Interrupted Time-Series Analysis

**Figure 2:**
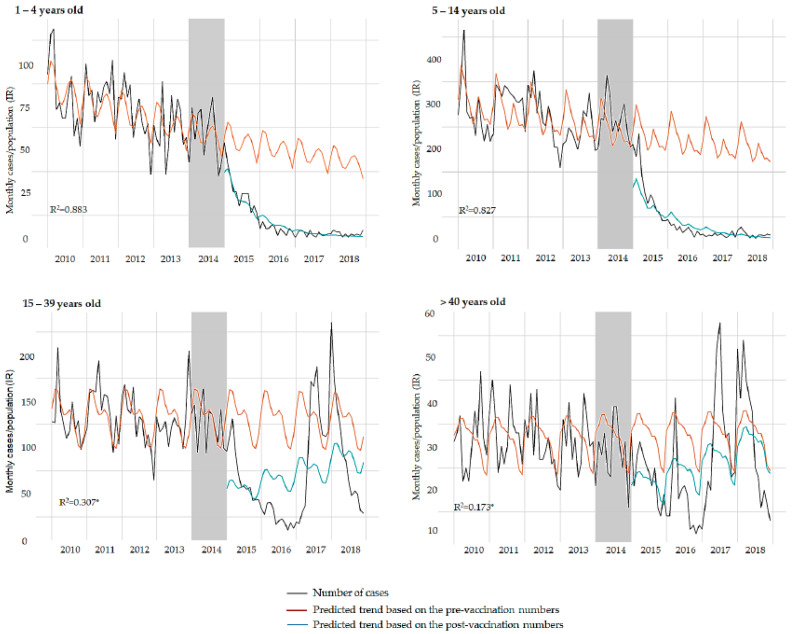
Trends in monthly number of hepatitis A cases over the study period for each age group. Grey bars represent the year of hepatitis A vaccine introduction (transition period). * Model affected by the outbreak.

Prior to introduction of the hepatitis A vaccine via the NIP there was already a downtrend reduction in hepatitis A cases in all age groups (Table 2, Figure 2). Overall, there was a yearly reduction of 3.9% in the hepatitis A incidence rate in the pre-vaccination period and a greater yearly reduction in the post-vaccination period (13.4%); however, the difference between the pre- and post-vaccination slopes was not statistically significant (*p* = 0.148). This suggests the vaccination did not result in a significant difference to the yearly reduction already observed in the pre-vaccination period, considering the entire country. Nevertheless, immediately after the vaccine introduction, the incidence rate drastically decreased across all age groups (*p* < 0.001). In fact, immediately following the vaccine introduction, there was a reduction of 67.1% on the hepatitis A incidence rate, when considering the entire population. 

In the stratified analyses per age-groups, the downward trend became more precipitated with vaccination effect compared to the counterfactual trend without vaccination effect. This was statistically different for age groups under 15 years old (*p* < 0.001). Indeed, the target-NIP population (1–4 years old) showed the greatest impact over the period after vaccination program, with a mean annual reduction of 67.6% in hepatitis A incidence rate (*p* < 0.001). Children 5–14 years old also presented a significant decreasing trend, with an annual reduction of 53.7% in hepatitis A incidence rate in the post-vaccination period. 

**Figure 3:**
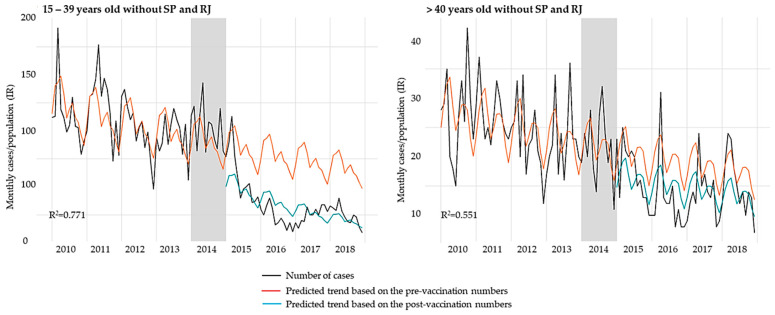
Trends in monthly number of hepatitis A cases over the study period for age groups 15–39 years old and ≥40 years old subtracting data from São Paulo and Rio de Janeiro. Grey bars represent the year of hepatitis A vaccine introduction (transition period).

In contrast, for subjects over 15 years, there was an increase in the predicted annual cases after the hepatitis A introduction in the NIP, with a mean annual rise of 17.4% and 9.5% in hepatitis A incidence rate for groups aged 15–39 years old and ≥40 years old, respectively. This trend suggested an outbreak, as was observed in the Southeast region, mainly in São Paulo and Rio de Janeiro states in 2017 [14]. To overcome the influence of the Southeast outbreak, we conducted a secondary time-series analysis excluding those states to project an outbreak-free scenario (Table 3, Figure 3). Without the outbreak influence, the post-vaccination period showed a downtrend in hepatitis A incidence rates for both 15–39 years old (–24.9%) and ≥40 years old (−8.4%) groups. The comparisons between pre- and post-vaccination trends were significantly different for the 15–39 years old age group (*p* < 0.001), but showed no difference for the over 40 years group (*p* = 0.911), indicating that the outbreak affected mainly the young adults. In this scenario, the vaccine effectively reduced the hepatitis A incidence rate in the overall population, with a significant difference in the pre- and post-vaccination trends (*p* < 0.001), and the downward trend becoming more precipitated in the post-vaccination period. 

The time-series model also allowed the prediction of the number of hepatitis A cases in the post-vaccination period if the hepatitis A vaccine had not been introduced via the NIP. Considering the entire population and based on the predicted cases of the pre- and post-vaccination period, the vaccination program prevented 14,468 hepatitis A cases between 2015 and 2018, even with the influence of the Southeast outbreak. When São Paulo and Rio de Janeiro were excluded from data analysis of individuals over 15 years old, the proportion of averted cases was slightly lower, with 13,008 cases prevented (Table 4). 

### 3.4. Fulminant Hepatitis Cases

**Figure 4:**
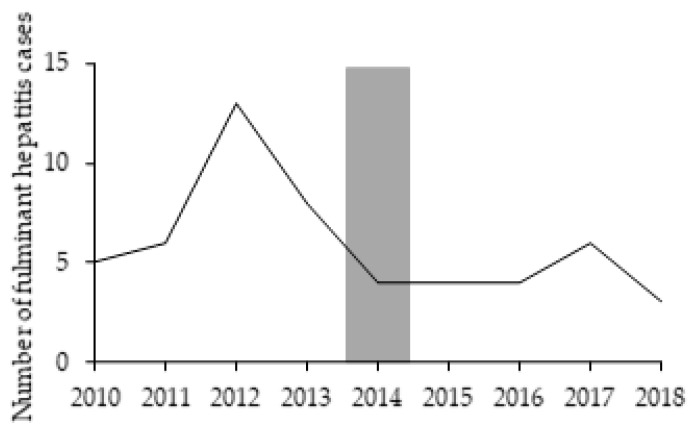
Overall fulminant hepatitis cases reported between 2010 and 2018. Grey bar represents the year of hepatitis A vaccine introduction (transition period).

During the pre-vaccination period, four cases of fulminant hepatitis were reported in the target population group (children aged 1–4 years old). After vaccine introduction no cases were reported (Table A2). After the hepatitis A vaccine introduction, the overall fulminant hepatitis cases/incidence halved, although no statistical difference was observed (*p* = 0.955) (Table 5). Despite this overall reduction, there was a peak in 2017. This was likely associated with the Southeast outbreak, since most cases occurred in individuals between 20 and 59 years old (Figure 4, Table A2). 

## 4. Discussion

To our knowledge, this is the first study using interrupted time-series analysis to evaluate the impact of introducing a hepatitis A vaccine into the NIP on hepatitis A incidence rates, in the Brazilian population. Previous studies have demonstrated important reductions of hepatitis A incidence in all regions of Brazil after the vaccine introduction using other methodologies [9,13,14,15]. We found a significant immediate reduction (level change) of hepatitis A incidence rates in the overall population as well as in the target population for vaccination. The reduction of incidence rates per year (slope) was accentuated in the post-vaccination period compared to pre-vaccination period among children under 14 years of age. Over the period of 2015–2018, we estimated that 14,468 hepatitis A cases were prevented in Brazil after the vaccine introduction. 

In Brazil, the inactivated hepatitis A vaccine was made universally available in mid-2014 as a single-dose vaccine for children aged 12–24 months and the vaccination coverage goal was set at 95% by the Ministry of Health. Since then, the national coverage goal has been achieved only once, in 2015, followed by a marked reduction in 2016 (Figure 1). Nevertheless, a sustained vaccination coverage from 65% to 85% was reported between 2017 and 2019 with a slightly increasing trend over the years in all national territories. The drop in hepatitis A vaccination coverage in 2016 was consistent throughout all vaccines included in the childhood immunization schedule, probably due to multiple factors including lack of health education promotion on the benefits of vaccination and the change of the NIP information system from consolidated doses applied to nominal register [13]. From 1994 until 2013, information on vaccine doses administered was provided by the municipalities administrators as aggregated data. Since 2014, the SI-PNI has gradually implemented an electronic national vaccination registry module with patient-level data, which depends on an adequate infrastructure and trained personal within the vaccination rooms of the municipalities to report the administered doses. 

In the North region, despite the low baseline vaccination coverage (Figure 1), we observed the greatest incidence decline within all regions. It represents the region with the lowest Human Development Index (HDI) in Brazil, with less access to, or availability of, education, health, water, and sanitation services. Only 55.4% of residents in the North region has access to water supply and 10.5% to sanitation services, contributing to a favorable environment for HAV transmission [16]. In this region, it was very clear how vaccination policy can be used as a tool for closing health disparities between populations. Conversely, in the Southeast region, which has the highest HDI with better access to water and sanitation services [16], the incidence rate in 2018 was higher than in 2010, even with sustained vaccination coverage >67% between 2014 and 2019. According to epidemiological reports [17], this increase was mainly observed as a temporal and geographical cluster of cases in São Paulo and Rio de Janeiro states among young male adults [14]. A secondary time-series analysis excluding the population affected by the outbreak demonstrated that the universal vaccination, paired with proper protection of adults with risk factors, could have reduced hepatitis A infection at a larger scale and a faster pace. 

Since February 2016, hepatitis A outbreaks in MSM populations have been reported in many parts of the world including the major cities of Latin America [3,18,19,20]. Molecular epidemiology shows similar HAV strains circulating among HAV-infected MSM, suggesting a distinct route of transmission through sexual contact [11]. Unlike Argentina, where vaccination is offered to the MSM population within the NIP, Brazil does not include this population among the individuals eligible to receive the vaccination. For this population, as well as for other individuals at substantial risk of contracting hepatitis A, the preferred regimen is the traditional two-dose schedule given 6–18 months apart [13]. It is expected that a greater reduction in hepatitis A cases will occur when those immunized via the current NIP eventually become young adults. However, an immediate inclusion of the MSM population in the Brazilian NIP would be more effective in preventing hepatitis A outbreaks and HAV transmission [21]. 

Compared to the results shown by Souto et al. [10], our methodology discounted the effect of the decreasing pre-vaccination trends and, as expected, showed somewhat smaller population impact. Our results complement findings from studies in Argentina of the impact of a single-dose vaccine in reducing HAV circulation and symptomatic hepatitis A cases. In Argentina, fulminant hepatitis and liver transplantation also reduced drastically in a short period of time [22,23,24]. In Brazil, although the overall fulminant hepatitis cases halved after the vaccine introduction, there was no statistical difference in the overall fulminant hepatitis incidence rate, likely due to low number of cases observed between 2010 and 2018. This difference between Argentina and Brazil was likely due to the seriousness of the baseline incidence in the former country. 

Traditionally, the two-dose vaccine is recommended for complete immunization, offering approximately 99% effectiveness and up to 20 years long-term immunogenicity in real-world studies and ≥30 years in modeling studies [25,26,27,28,29]. However, the 2012 WHO position paper on hepatitis A vaccines stated that national immunization programs may consider inclusion of single-dose inactivated hepatitis A vaccines in childhood immunization schedules [10] based on experience in Argentina [30]. This recommendation was followed by other low and middle income countries of Latin America as Brazil, Chile and Colombia introduced hepatitis A vaccination in their childhood immunization schedules [31]. Other countries outside Latin America have shown similar positive effects after the use of a single-dose hepatitis A vaccine, such as Russia [32]. Our study results mirror high immunogenicity acquired after the first dose of hepatitis A vaccine. Clinical trials conducted in the United States with inactivated hepatitis A vaccine showed protective antibody concentration in 92% of toddlers after a single dose and 100% after two doses [33]. Longer term studies from Argentina have shown protective antibodies against HAV in 97.0% and 87.6% of Argentinean children after 9 and 11 years of the introduction, respectively [34]. In Brazil, a preliminary study demonstrated that 93.6% of children seroconverted 30 days after the immunization [15]. Further long-term assessments in the Brazilian population are required to confirm the seroprotective effect of a single-dose HAV vaccine. 

The Global Burden of Disease (GBD) project estimates that hepatitis A resulted in 2.35 million (95% UI 1.70–3.09) global disability-adjusted life year (DALYs) in 2019 and was responsible for 53.9% (42.9–63.6) of acute hepatitis DALYs [35]. In Americas WHO region, Latin America concentrated the highest burden of the disease. The findings of this study can provide useful information for policy makers in Latin America, a region characterized by middle-income countries with a mix of subpopulations with intermediate and low seroprevalences [36], leaving a large proportion of adolescents and adults susceptible. Our results reassure the effectiveness of a single dose schedule and how hepatitis A national childhood immunization may be a proper disease prevention strategy in Latin America and other countries with similar epidemiological profile.

This study has some limitations innate to the database used to source epidemiological data. The hepatitis A incidence rate considered only the cases that were reported through SINAN database, either those symptomatic or those identified through routine assessments, considering both the public and private healthcare systems. Therefore, the number of hepatitis A cases could be underestimated. It is also important to emphasize that the vaccination coverage reported here was based on aggregated data provided by municipal administrators regarding total doses administrated annually and population census estimates. Thus, some variations in the values are expected due to the inherent biases in the methodology applied to estimate vaccination coverage. Finally, as this is a retrospective study based solely on data from government databases, the validity of data enclosed could not be verified through medical records. However, data is assumed to be both sensitive and specific, as collected as part of notifiable disease reporting and requiring a combination of laboratory and clinical criteria. Therefore, it is expected that this study represents a real-world scenario in Brazil. 

## 5. Conclusions

Our study provided additional evidence that a one-dose inactivated hepatitis A vaccination is effective in controlling hepatitis A at a national level, with the target-NIP population (children aged 1–4 years) receiving the greatest benefit. Policy makers in other Latin American countries may utilize our findings to tailor their universal vaccination strategies for hepatitis A. Additional assessments and continued monitoring are needed to evaluate long-term protection after a single dose in the Brazilian population. 

## Figures and Tables

**Table 1:** Comparative analysis of incidence rates before and after the hepatitis A introduction in the National Immunization Program, according to Brazilian geographic region.

	Pre-Vaccination Period	Post-Vaccination Period	Variation	95% CI		*p*-Value
Brazil	3.18	0.87	−2.31	0.33	4.28	0.022
North	13.69	3.04	−10.66	−6.65	−14.66	<0.0001
Northeast	3.67	0.42	−3.25	− 5.23	−1.26	0.001
Midwest	3.06	0.66	−2.40	−4.29	−0.51	0.013
South	1.88	0.52	−1.36	−2.88	0.16	0.079
Southeast	1.22	0.88	−0.34	−1.76	1.08	0.641

**Table 2:** Time-series model estimates of the impact of hepatitis A vaccination on yearly cases number, by age group.

	Variation (%)	2.5% Percentile	97.5% Percentile	*p*-Value
Overall				
Immediate effect ^a^	−67.1	−76.4	−54.2	<0.001
Trend without vaccination effect ^b^	−3.9	−13.1	6.3	0.4391
Trend with vaccination effect ^c^	−13.4	−21.9	−4.1	0.0056
Comparison of pre-post trends ^d^	-	-	-	0.148
<12 months of age				
Immediate effect ^a^	−54.0	−69.8	−30.0	<0.001
Trend without vaccination effect ^b^	−0.4	−0.8	9.0	0.9465
Trend with vaccination effect ^c^	−34.5	-46.2	25.3	<0.001
Comparison of pre-post trends ^d^	-	-	-	<0.001
1–4 years old				
Immediate effect ^a^	−52.5	−61.3	−41.7	<0.001
Trend without vaccination effect ^b^	−7.7	−11.8	−3.4	<0.001
Trend with vaccination effect ^c^	−67.6	−71.7	−62.8	<0.001
Comparison of pre-post trends	-	-	-	<0.001
5–14 years old				
Immediate effect ^a^	−57.1	−66.9	−44.3	<0.001
Trend without vaccination effect ^b^	−3.4	−10.5	4.2	0.3651
Trend with vaccination effect ^c^	−53.7	−58.0	−49.1	<0.001
Comparison of pre-post trends ^d^	-	-	-	<0.001
15–39 years old				
Immediate effect ^a^	−62.7	−75.1	−44.1	<0.001
Trend without vaccination effect ^b^	−0.7	−12.2	12.2	0.914
Trend with vaccination effect ^c^	17.4	3.7	32.9	0.0114
Comparison of pre-post trends ^d^	-	-	-	0.057
≥40 years old				
Immediate effect ^a^	−43.3	−56.1	−26.8	<0.001
Trend without vaccination effect ^b^	−1.9	−9.2	5.8	0.6154
Trend with vaccination effect ^c^	9.5	1.2	18.6	0.024
Comparison of pre-post trends ^d^	-	-	-	0.045

^a^ Immediate effect: level change between pre- (trend without vaccination effect) and post-vaccination (trend with vaccination effect) periods. ^b^ Trend without vaccination effect: mean annual variation (slope) in hepatitis A cases during pre-vaccination period (2010–2013). ^c^ Trend with vaccination effect: mean annual variation (slope) in hepatitis A cases during post-vaccination period (2015–2018). ^d^ Comparison between trend without (counterfactual) and with vaccination effect.

**Table 3:** Time-series model estimates of the impact of hepatitis A vaccination on yearly cases number for age groups 15–39 years old and ≥40 years old subtracting data from São Paulo and Rio de Janeiro.

Without São Paulo and Rio de Janeiro Data	Variation (%)	2.5% Percentile	97.5% Percentile	*p*-Value
Overall				
Immediate effect ^a^	−63.9	−71.3	−54.7	<0.001
Trend without vaccination effect ^b^	−6.4	−12.6	0.2	0.0594
Trend with vaccination effect ^c^	−36.8	−41.4	−32.1	<0.001
Comparison of pre-post trends ^d^	-	-		<0.001
15–39 years old				
Immediate effect ^a^	−57.4	−65.8	−46.9	<0.001
Trend without vaccination effect ^b^	−7.5	−13.2	−1.4	0.016
Trend with vaccination effect ^c^	−24.9	−30.3	−19.1	<0.001
Comparison of pre-post trends ^d^	-	-	-	<0.001
≥40 years old				
Immediate effect ^a^	−48.0	−57.7	−36.0	<0.001
Trend without vaccination effect ^b^	−7.9	−13.1	−2.5	0.0049
Trend with vaccination effect ^c^	−8.4	−14.8	−1.7	0.0151
Comparison of pre-post trends ^d^	-	-	-	0.911

^a^ Immediate effect: level change between pre- (trend without vaccination effect) and post-vaccination (trend with vaccination effect) periods. ^b^ Trend without vaccination effect: mean annual variation (slope) in hepatitis A cases during pre-vaccination period (2010–2013). ^c^ Trend with vaccination effect: mean annual variation (slope) in hepatitis A cases during post-vaccination period (2015–2018). ^d^ Comparison between trend without (counterfactual) and with vaccination effect.

**Table 4:** Number of observed, predicted, and averted hepatitis A cases in the post-vaccination period (2015–2018), by age group. A subanalysis was conducted for subjects over 15 years old, subtracting São Paulo (SP) and Rio de Janeiro (RJ) cases.

	Observed	Predicted	Averted Cases	
			Median	Percentiles (2.5%; 97.5%)
Entire population				
<12 months of age	88	404	316	288; 336
1–4 years old	423	2375	1952	1855; 2023
5–14 years old	1925	10,427	8502	8062; 8857
15–39 years old	3495	6302	2807	2488; 3025
≥40 years old	1255	1575	320	262; 371
Total	7186	21,654	14,468	13,395; 15,138
Without São Paulo and Rio de Janeiro data				
15–39 years old	1589	3632	2043	1846; 2160
≥40 years old	708	910	202	165; 228
Total	4733	17,741	13,008	12,214; 13,487

**Table 5:** Fulminant hepatitis number of cases and incidence during the period of the study.

	Pre-Vaccination Period	Post-Vaccination Period	Variation	95% Conf. Interval		*p*-Value
Total fulminant hepatitis cases	32	17	15	-	-	-
Mean incidence rates of fulminant hepatitis	0.004	0.002	0.002	−0.08	0.075	0.955

## Data Availability

All data reported were obtained from DATASUS site (http://www2.datasus.gov.br/DATASUS/index.php (accessed on 15 April 2021)).

## Appendix A

**Table A1:** Hepatitis A cases reported between 2010 and 2018 (including the year of 2014) according to age group and Brazilian geographic region.

	Confirmed Hepatitis A Cases between 2010 and 2018	Percentage of Cases
Age group		
<12 months	615	1.6%
12–24 months	307	0.8%
2–4 years old	4371	11.5%
5–9 years old	11,299	29.6%
10–14 years old	7141	18.7%
15–19 years old	3841	10.1%
20–39 years old	7497	19.6%
40–59 years old	2217	5.8%
60–64 years old	268	0.7%
65–69 years old	255	0.7%
70–79 years old	284	0.7%
80+ years old	92	0.2%
Brazilian region		
Midwest	2742	7.2%
North	13,796	36.1%
Northeast	10,833	28.4%
South	2886	7.6%
Southeast	7930	20.8%

**Table A2:** Number of fulminant hepatitis cases reported between 2010 and 2018 according to age group.

	Fulminant Hepatitis	
	Number of Cases	Incidence
2010		
<12 months	-	-
12–24 months	-	-
2–4 years old	-	-
5–9 years old	1	0.006
10–14 years old	2	0.011
15–19 years old	1	0.006
20–39 years old	-	-
40–59 years old	1	0.002
60–64 years old	-	-
65–69 years old	-	-
70–79 years old	-	-
>80 years old	-	-
2011		
<12 months	-	-
12–24 months	-	-
2–4 years old	1	0.011
5–9 years old	-	-
10–14 years old	-	-
15–19 years old	-	-
20–39 years old	3	0.005
40–59 years old	2	0.004
60–64 years old	-	-
65–69 years old	-	-
70–79 years old	-	-
>80 years old	-	-
2012		
<12 months	1	0.034
12–24 months	-	-
2–4 years old	3	0.034
5–9 years old	3	0.019
10–14 years old	-	-
15–19 years old	1	0.006
20–39 years old	1	0.002
40–59 years old	4	0.009
60–64 years old	-	-
65–69 years old	-	-
70–79 years old	-	-
>80 years old	-	-
2013		
<12 months	-	-
12–24 months	-	-
2–4 years old	-	-
5–9 years old	-	-
10–14 years old	3	0.018
15–19 years old	-	-
20–39 years old	3	0.005
40–59 years old	2	0.004
60–64 years old	-	-
65–69 years old	-	-
70–79 years old	-	-
>80 years old	-	-
2014		
<12 months	-	-
12–24 months	-	-
2–4 years old	1	0.011
5–9 years old	-	-
10–14 years old	1	0.006
15–19 years old	-	-
20–39 years old	2	0.003
40–59 years old	-	-
60–64 years old	-	-
65–69 years old	-	-
70–79 years old	-	-
>80 years old	-	-
2015		
<12 months	1	0.033
12–24 months	-	-
2–4 years old	-	-
5–9 years old	-	-
10–14 years old	1	0.006
15–19 years old	-	-
20–39 years old	-	-
40–59 years old	2	0.004
60–64 years old	-	-
65–69 years old	-	-
70–79 years old	-	-
>80 years old	-	-
2016		
<12 months	1	0.034
12–24 months	-	-
2–4 years old	-	-
5–9 years old	-	-
10–14 years old	-	-
15–19 years old	-	-
20–39 years old	1	0.001
40–59 years old	-	-
60–64 years old	-	-
65–69 years old	1	0.016
70–79 years old	1	0.013
>80 years old	-	-
2017		
<12 months	-	-
12–24 months	-	-
2–4 years old	-	-
5–9 years old	-	-
10–14 years old	-	-
15–19 years old	-	-
20–39 years old	3	0.004
40–59 years old	2	0.004
60–64 years old	-	-
65–69 years old	1	0.015
70–79 years old	-	-
>80 years old	-	-
2018		
<12 months	-	-
12–24 months	-	-
2–4 years old	-	-
5–9 years old	-	-
10–14 years old	-	-
15–19 years old	-	-
20–39 years old	2	0.003
40–59 years old	1	0.002
60–64 years old	-	-
65–69 years old	-	-
70–79 years old	-	-
>80 years old	-	-
